# Impact of projected sea surface temperature biases on tropical cyclones projections in the South Pacific

**DOI:** 10.1038/s41598-020-61570-6

**Published:** 2020-03-16

**Authors:** C. Dutheil, M. Lengaigne, M. Bador, J. Vialard, J. Lefèvre, N. C. Jourdain, S. Jullien, A. Peltier, B. Sultan, C. Menkès

**Affiliations:** 1 ENTROPIE (UMR 9220), IRD, Univ. de la Réunion, CNRS, Nouméa, Nouvelle-Calédonie; 2 LOCEAN-IPSL, Sorbonne Universités, UPMC, Université Paris 06, CNRS-IRD-MNHN, Paris, France; 3 ARC Centre of Excellence for Climate Extremes and Climate Change Research Centre, School of BEES, University of New South Wales, Sydney, New South Wales Australia; 4 Univ. Grenoble Alpes, CNRS, IRD, G-INP, IGE, Grenoble, France; 5 Ifremer, Univ. Brest, CNRS, IRD, Laboratoire d’Océanographie Physique et Spatiale (LOPS), IUEM, Plouzané, France; 6 Météo France, Nouméa, New Caledonia; 7 ESPACE-DEV, Univ Montpellier, IRD, Univ Guyane, Univ Reunion, Univ Antilles, Univ Avignon, Avignon, France

**Keywords:** Atmospheric science, Climate change

## Abstract

Climate model projections generally indicate fewer but more intense tropical cyclones (TCs) in response to increasing anthropogenic emissions. However these simulations suffer from long-standing biases in their Sea Surface Temperature (SST). While most studies investigating future changes in TC activity using high-resolution atmospheric models correct for the present-day SST bias, they do not consider the reliability of the projected SST changes from global climate models. The present study illustrates that future South Pacific TC activity changes are strongly sensitive to correcting the projected SST changes using an emergent constraint method. This additional correction indeed leads to a strong reduction of the cyclogenesis (−55%) over the South Pacific basin, while no statistically significant change arises in the uncorrected simulations. Cyclogenesis indices suggest that this strong reduction in the corrected experiment is caused by stronger vertical wind shear in response to a South Pacific Convergence Zone equatorward shift. We thus find that uncertainty in the projected SST patterns could strongly hamper the reliability of South Pacific TC projections. The strong sensitivity found in the current study will need to be investigated with other models, observational constraint methods and in other TC basins in order to assess the reliability of regional TC projections.

## Introduction

Tropical Cyclones (TCs) are among the most devastating atmospheric phenomena, with drastic socio-economic consequences in coastal regions. Accurately projecting TC activity changes in a global warming context is hence a crucial challenge. The last Intergovernmental Panel on Climate Change (IPCC) assessment^1^ indicates “that it is likely that global frequency of TCs will either decrease or remain essentially unchanged”, and that an increase in the frequency of most intense TCs for the 21^st^ century is “more likely than not”. This assessment^1^ also stresses the low-confidence level in basin-scale TC projections, given the contrasting results from existing studies. Therefore, TC projections are still uncertain despite their crucial importance.

The future climate response to anthropogenic forcing is generally derived from the analysis of simulations from the Coupled Model Intercomparison Project (CMIP) database^2^. CMIP models are generally unable to realistically simulate TC activity^3^ due to their coarse atmospheric horizontal resolutions (100 to 200 km). A downscaling technique is hence commonly used to provide insights on the future changes in TC activity. It consists in experiments at higher horizontal resolution (10 to 50 km) performed with regional or global atmospheric models forced by CMIP5 projections at their boundaries (SST for global models; SST and lateral atmospheric boundary conditions for regional models). Such experiments adequately resolve TCs^4,5^.

CMIP5 models however exhibit substantial biases in their present-day climatologies, including an underestimated zonal SST gradient in the equatorial Atlantic^6^, an equatorial Pacific cold tongue that penetrates too far westward^7–10^, and a tendency for the South Pacific Convergence Zone (SPCZ) to be too zonal and to extend too far eastward^11,12^. Forcing a high-resolution atmospheric model with those biased boundary conditions induces systematic biases on the simulated TC climatology, and probably hampers the future projections^13,14^. A commonly-used strategy to alleviate this shortcoming is to prescribe “climate change anomalies”, i.e. atmospheric simulations are forced by observed present-day SSTs, onto which the anomalous ensemble mean sea surface warming pattern from CMIP5 projections is added^15^. This approach is sometimes referred to as pseudo-global warming (PGW) downscaling, although this term was also used to describe the application of more ad-hoc anomalies^16^. Strong uncertainties in regional projections of future TC activity also arise from the various projected SST warming patterns for a given scenario in the CMIP database^17–21^. For instance, Murakami *et al*.^18^ showed a significant variation in future TC number per basin depending on the CMIP3 SST pattern used. This raises the issue of the sensitivity of TC projections to the projected SST change does not correct.

**Figure 1:**
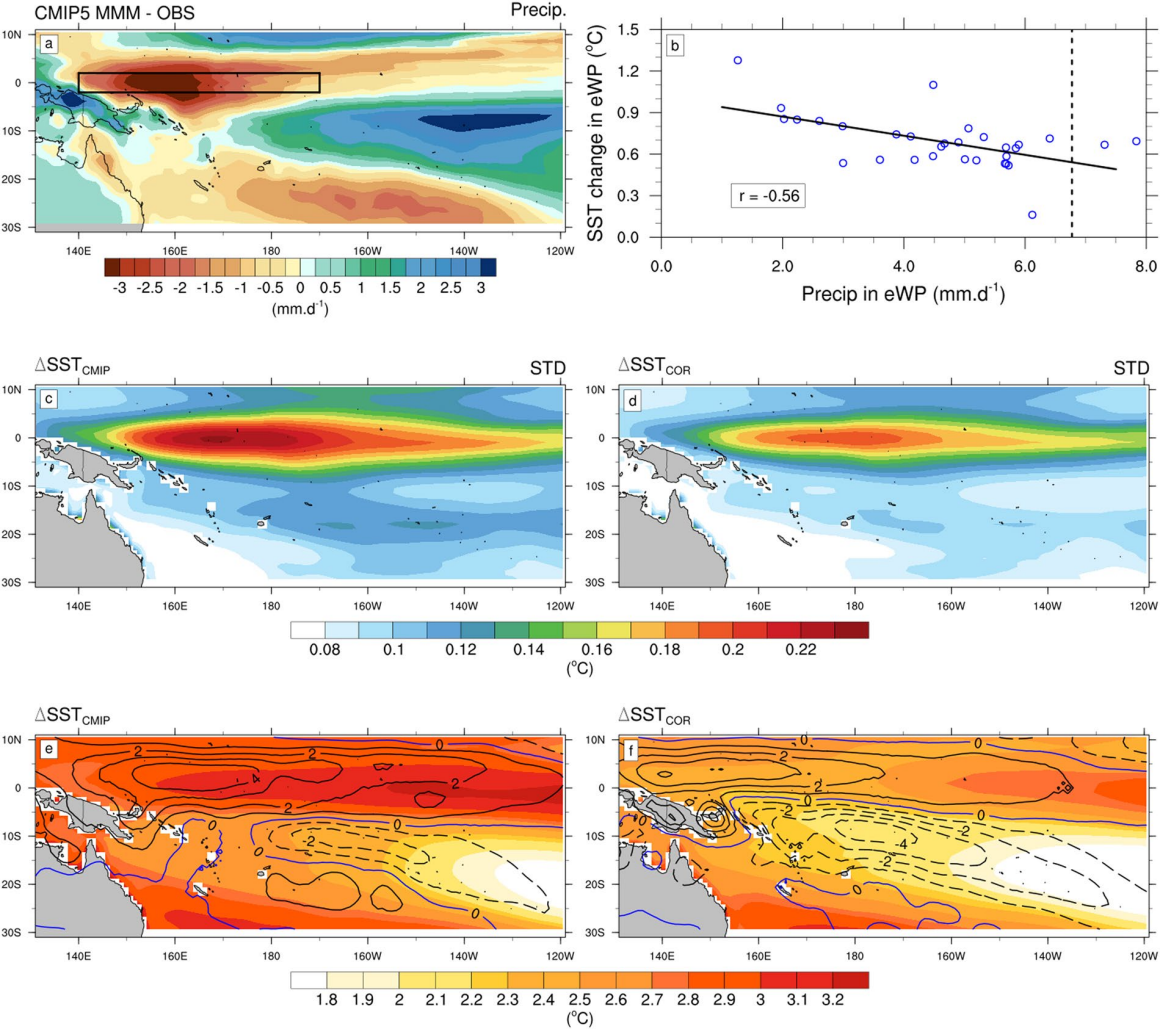
Top: (**a**) Annual multi-model mean CMIP5 of precipitation bias (in mm.day^−1^) relative to observations (CMAP). (**b**) Relationships between the annual mean of SST projected changes (°C) and the historical mean precipitation (mm.day^−1^) in the equatorial western Pacific [140°E-170°W;2°S-2°N] among 31 CMIP5 models. The inter-model correlation (**r**) is shown at the bottom-left. The dashed line on the panels b denotes the observed mean precipitation in the equatorial western Pacific. Middle: Annual mean of inter-model standard deviations of (**c**) uncorrected and (**d**) corrected SST changes (in °C). Bottom: DJF climatology (shading, in °C) of (**e**) ΔSST_CMIP_ and (**f**) ΔSST_COR_. The contours represent the DJF climatology of precipitation (in mm.d^−1^) changes between (**e**) CC and PD, (**f**) COR and PD simulations. The dashed lines indicate negative values, while the solid lines indicate positive values.

**Figure 2:**
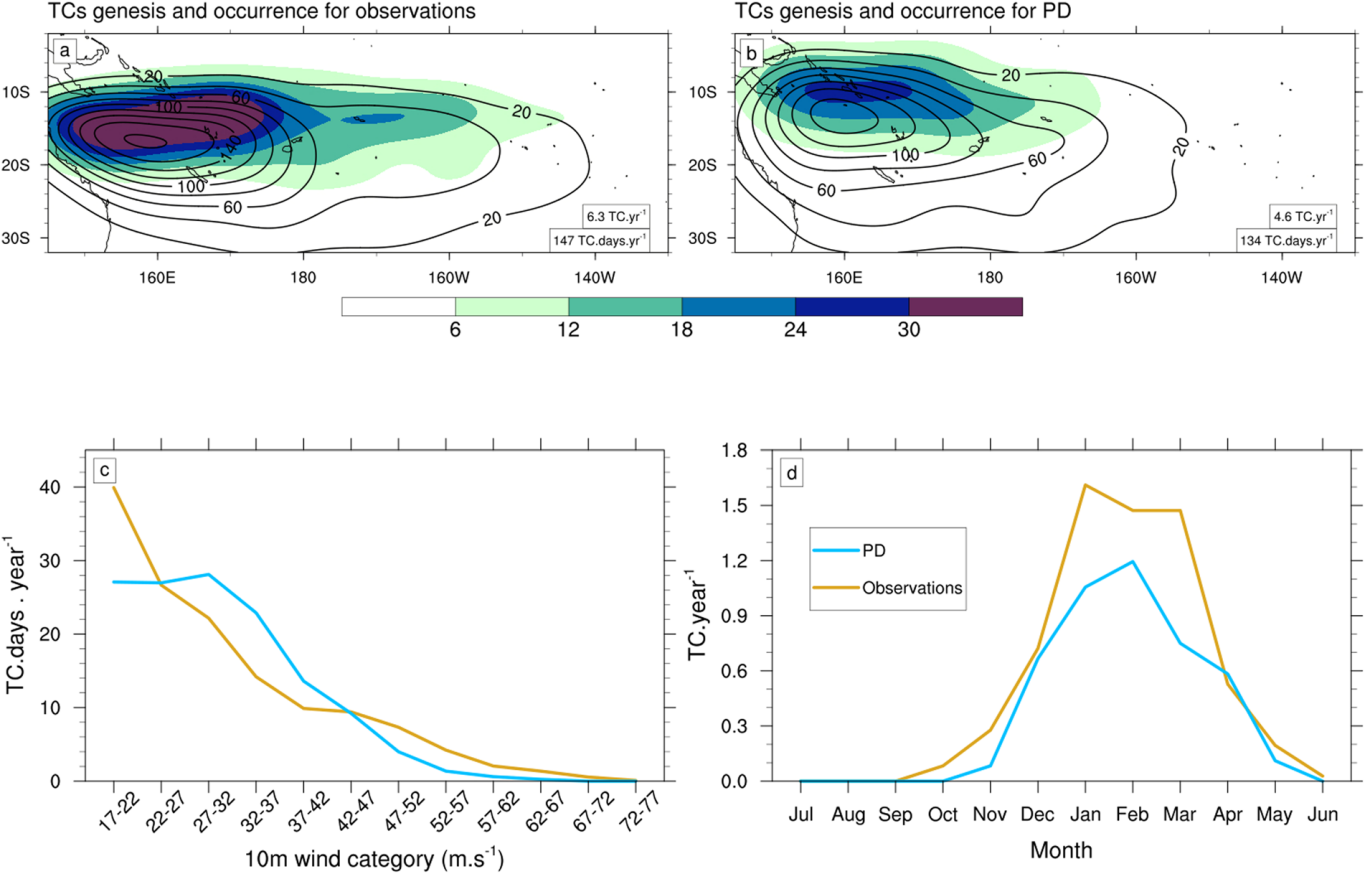
Top: Probability Distribution Functions (PDFs) of TCs genesis (shading) and occurrence (contour lines) for (**a**) observations (IBTrACS) and (**b**) PD. To generate PDFs, we compute anisotropic Gaussian functions, with an associated standard deviation in meridional and zonal directions respectively of 2.5° and 5°. The area-averaged annual mean values over the plotted spatial domain of TC genesis and occurrence are shown in the corresponding panels. Bottom: (**c**) Annual mean frequency of TC occurrence (in TC.days.year^−1^) as a function of the maximum 10-m wind speed (in m.s^−1^) and (**d**) the seasonal cycle of monthly TC genesis number (in TC.year^−1^) for observations (gold) and PD (blue).

Using a multi-model ensemble mean projected SST change as the one displayed in Fig. 1e reduces the projected SST errors as it largely cancels the model-dependent part of the errors^22^. Applying the PGW approach with the multi-model ensemble mean projected SST change therefore improves the reliability of projections. This method however does not correct for the systematic biases (*i.e*. common to all models) in the SST response to anthropogenic forcing. A way to circumvent this issue is to correct the projected warming using a statistical relationship between present-day biases and projected changes, referred to as “observational constraint” or “emergent constraint”^23,24^. Li *et al*.^25^ for instance identified a strong relation between the present-day western equatorial Pacific dry bias and SST projections in CMIP5 models that can be used to correct and reduce the uncertainty in those SST projections. Using the PGW downscaling approach, Dutheil *et al*.^26^ further compared regional South Pacific (SP) climate change simulations forced by two different SST surface boundary conditions: the uncorrected CMIP5 ensemble mean SST and its “corrected” counterpart based on the Li *et al*.^25^ emergent constraint (see Methods). Their results indicate a large sensitivity of the future SP rainfall pattern to the projected SST pattern, with a considerably larger future SPCZ drying in the corrected simulations than in the uncorrected ones, due to altered SST gradients changing the circulation and humidity convergence. The SPCZ is also the breeding ground of the SP TCs (6 to 7 per year on average; Fig. 2a) raising the question of how such TC activity is impacted by those projected SST patterns.

The South Pacific (SP) region hosts thousands of low-lying islands, which are very vulnerable to TCs^27^. Those islands are also highly vulnerable to climate change, in particular to sea-level-rise^28^, and rainfall changes^27^. An increase in TC intensity or occurrence combined with rising sea level is expected to increase storm surge risks^29^. In this context, it is critical to describe the future regional changes in TC characteristics in the SP. Because of the uncertainties in TC records, the strong influence of internal variability on TC activity, and the modulation by the El Niño-Southern Oscillation (ENSO)^30–32^, observed historical long-term trends for SP TCs vary considerably from one study to another, ranging from a strong decrease in TC occurrence East of Australia^33^ to no significant changes^34,35^. Numerous numerical studies assessed future changes in TC activity over the SP region using a PGW approach^4,17–19,36–43^. All but one^41^ have reported a decrease in SP TC frequency under future climate. The amplitude of this decrease however varies widely across these studies, ranging from 10%^17^ to 60%^43^. These models also simulate a consistent intensification of TC-related precipitation^42,44,45^. The change in frequency of intense TCs is less consensual, with some studies indicating an increase^41,43^, and others indicating a decrease^4,18,38,40^.

This raises the question of whether and to what extent projected TC changes are also sensitive to the CMIP5 ensemble mean projected SST changes. The present study explores the sensitivity of changes in TC activity to correcting the CMIP5 ensemble mean projected SST changes using an emergent constraint method. To that end, we use a similar WRF model configuration than the one used in Dutheil *et al*.^26^, except that it includes a two-way nested domain at 21 km located over the Southwest Pacific that allows a realistic representation of TCs. We then perform three simulations: one present-day (labelled PD) simulation, and two climate-change simulations forced with the same boundary forcing as the one used in Dutheil *et al*.^26^: a multi-model uncorrected CMIP5 SST warming pattern for the first climate-change simulation (labelled CC), and an emergent-constraint-corrected CMIP5 SST warming pattern for the second climate-change simulation (labelled COR). All simulations use the PGW approach: the projected SST change is added on top of present-day SST conditions to avoid issues associated with present-day SST biases in CMIP5. Using that numerical framework, we also explore the mechanisms responsible for changes in TC projections when correcting the projected SST warming and the sensitivity of our results to the model parameterizations with two WRF model physics (labelled ZM and BMJ). Details about the regional model configuration and experimental designs are provided in the Methods section.

## Results

### Projected SST change correction

Li *et al*.^8,25^ showed that the typical “cold tongue bias” in the CMIP models is associated with insufficient mean precipitation and clouds over the western Pacific warm pool (Fig. 1a), which results in an underestimation of the convective feedback and an excessive SST warming response in the equatorial western Pacific. To illustrate this relationship, Fig. 1b shows the scatterplot between the historical rainfall bias and the projected SST change (ΔSST), averaged over the equatorial western Pacific across the 31 CMIP5 models (this analysis is similar to the analysis shown in Fig. 2 of Dutheil *et al*.^26^). The significant correlation between these two variables (−0.56, p-value < 0.001) indicates that the stronger the present-day dry bias (Fig. 1a), the stronger the projected ΔSST in this region. This linear relationship is further used to correct the ΔSST pattern projected by CMIP5 models (see Method section). The corrected CMIP5 ΔSST_COR_ exhibits a weaker warming in the South and equatorial western Pacific (Fig. 1e *vs* 1f), which significantly impacts the zonal and meridional SST gradients in the SP region. This correction method allows to strongly reduce the inter-model variance of the projected SST changes (Fig. 1c vs 1d), which indicates that the bias correction method improves the reliability of the projected SST changes. As detailed in Dutheil *et al*.^26^, forcing our model with these uncorrected and corrected CMIP5 ensemble mean SST warming patterns results in a large sensitivity of the future SP rainfall pattern to the projected SST pattern with a considerably larger southwestern Pacific rainfall reduction when correcting projected SST changes (contours in Fig. 1e *vs* 1f).

### Projected TC changes

The observed SP TC genesis is maximum northeast of Australia with the region of maximum TC occurrence shifted by 3° southward relative to that of the TC genesis pattern (Fig. 2a; see Method Section for TC tracking method description). The two-way nested domain at 21 km included in our model configuration allows the PD simulation to capture reasonably well this climatological TC pattern (Fig. 2a,b). The PD TC genesis and occurrence are however a few degrees closer to the equator than in the observations. TC genesis in PD (4.6 TC.year^−1^) is also underestimated compared to observations (6.3 TC.year^−1^), while TC occurrence is better captured (134 TC.days.year^−1^ in PD compared to 147 TC.days.year^−1^ in observations). Simulated TC intensity categories reasonably follow observed frequency distribution (Fig. 2c). The strongest simulated TC reaches 65 m.s^−1^ compared to 79 m.s^−1^ in the observations. PD also exhibits more frequent moderate TC intensity between 27 and 42 m.s^−1^ and less frequent intense (47 to 67 m.s^−1^) and weak TCs (lower than 22 m.s^−1^) compared to observations. The seasonal evolution of the TC density in PD agrees well with observations, with a cyclonic season extending from October to June and peaking in the early calendar year (January-February-March, JFM). The location of cyclogenesis is modulated by ENSO, and observations show that part of TC genesis moves eastward by several tens of degrees during El Niño years, and southward during La Niña years. These interannual displacements are well represented in our PD simulation (Fig. S1). These analyses indicate that the main SP TC characteristics are simulated reasonably well in PD.

**Figure 3:**
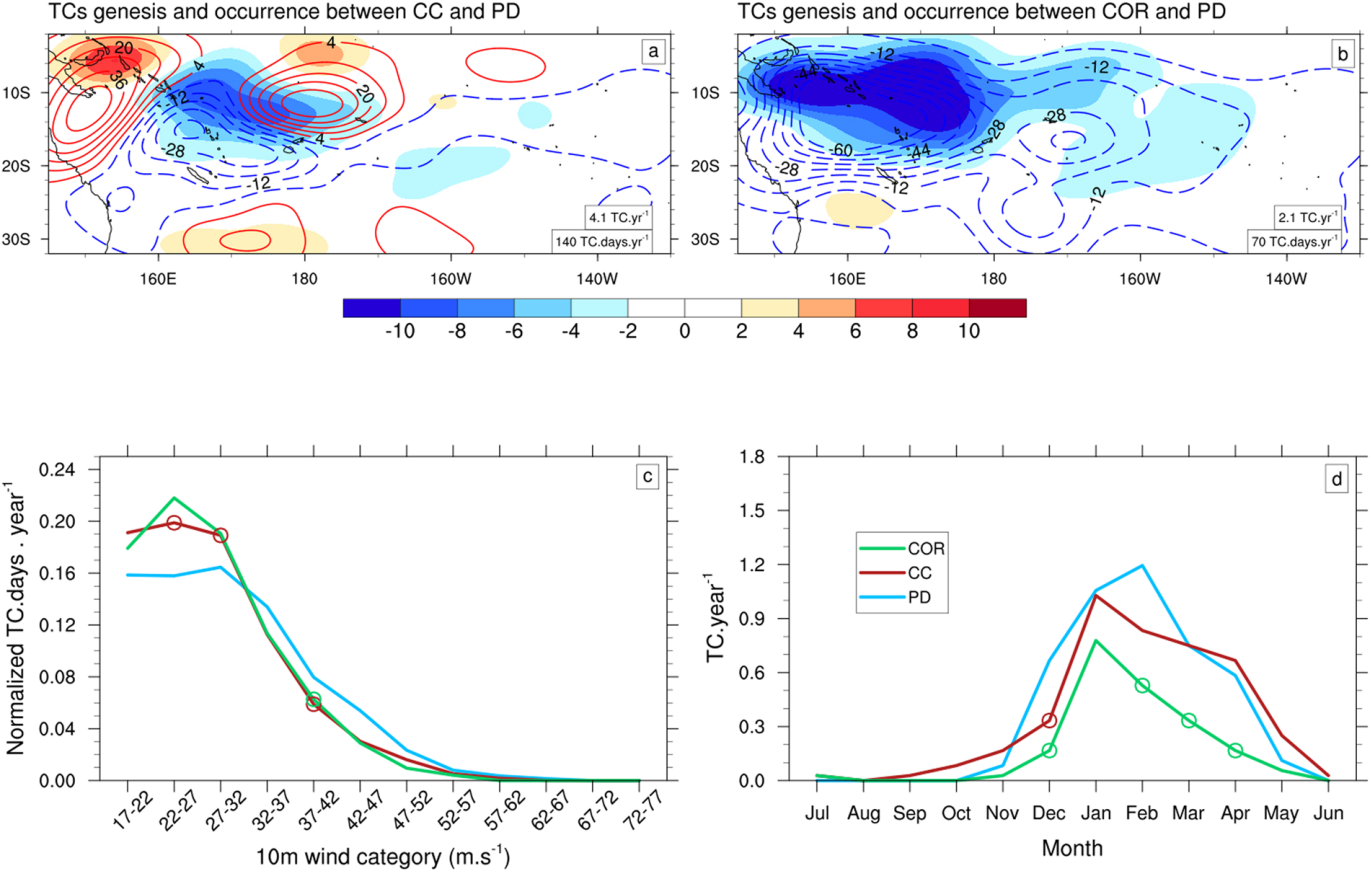
Top: PDFs of TCs genesis (shading) and occurrence (contour lines) between (**a**) CC and PD and (**b**) COR and PD. The values of annual mean TC genesis and occurrence are shown in the corresponding panels. Bottom: (**c**) Annual mean frequency of TC occurrence (in TC.days.year^−1^) as a function of the maximum 10-m wind speed (in m.s^−1^) and (**d**) the seasonal cycle of monthly TC genesis number (in TC.year^−1^) for PD (blue), CC (red) and COR (green). The circles on the lines indicate statistically significant future changes at 90% confidence level.

In CC, there is a non-significant (Mann–Whitney–Wilcoxon test, p-value = 0.7) and modest decrease (−11%) in TC genesis count and a non-significant increase in average TC occurrence frequency (+4%) over the SP basin (Fig. 3a,c,d), resulting from an increase of TC lifetime (+17%). CC displays a significant decrease in TC intensities (Fig. 3c), with a 23% increase of weak TCs (lower than 32 m.s^−1^), and a 33% reduction of stronger TCs (higher than 32 m.s^−1^). The seasonal cycle is not significantly changed (Fig. 3d), except for December, which exhibits a significant 50% decrease.

Correcting the projected SST warming pattern considerably impacts the projected TC activity (Fig. 3b) with a significant decrease in TC genesis of −55% (p-value = 1.10^–4^) in COR compared to −11% in CC. Unlike CC, COR displays a large −48% TC occurrence decrease. This decrease occurs rather homogenously over most of the SP domain (Fig. 3b) and the entire TC season (Fig. 3d). In contrast, the emergent constraint correction only slightly affects the projection of TC intensity distributions (Fig. 3c), with slightly more frequent weak TCs and less frequent strong TCs, although these changes are not significant relative to those in CC.

### Related mechanisms

**Figure 4:**
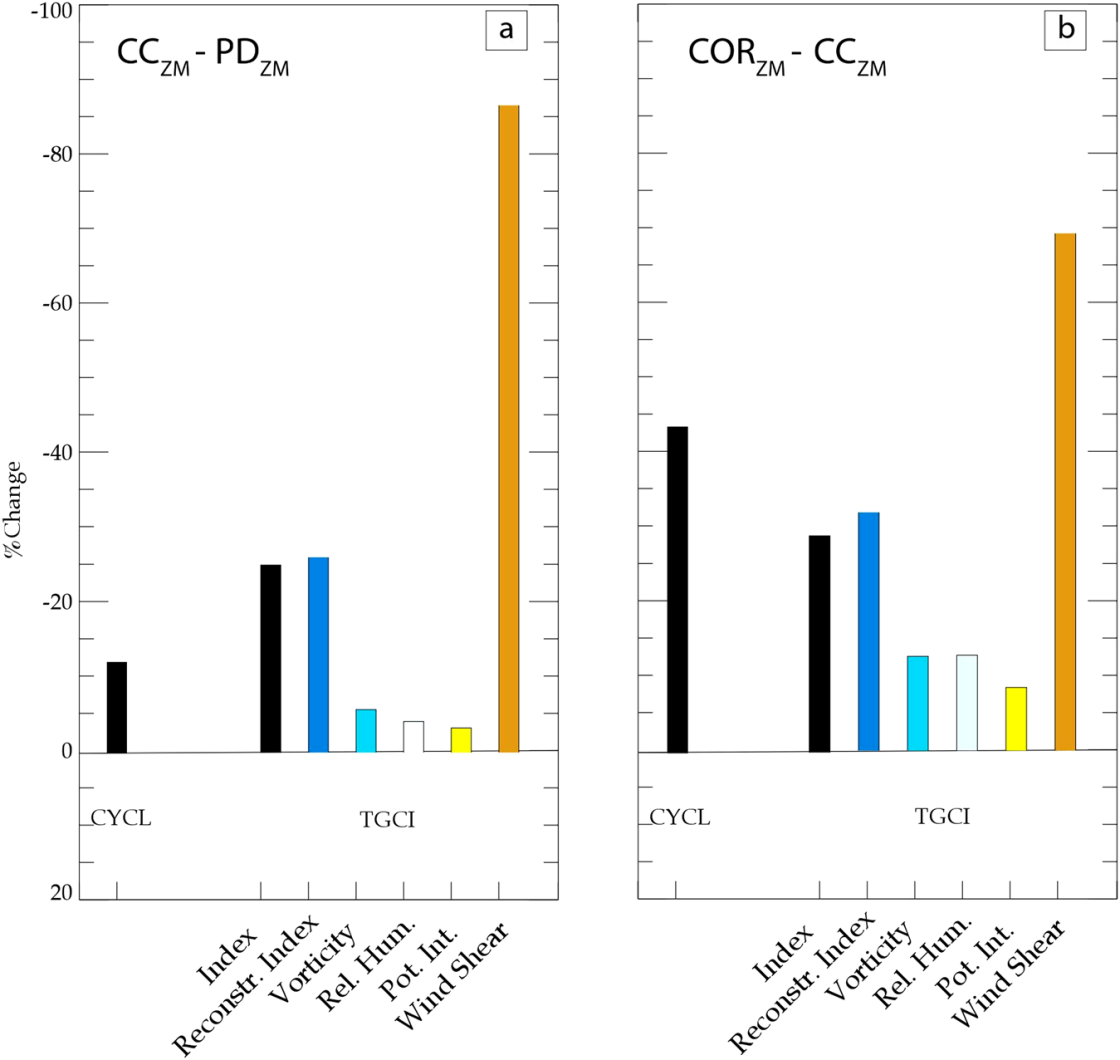
(**a**) Barplot of % of changes in TCs genesis between CC and PD and their contributing factors from the TCGI analysis (in %): TC genesis change (first bar) evaluated using the direct tracking method, TCGI change (second bar), reconstructed TCGI changes from the Taylor expansion analysis (third bar) and TCGI changes induced by each individual factors (fourth to seventh bar). (**b**) Same as (**a**) but for the changes between COR and CC. The individual factors (fourth to seventh bar) do not sum up to the reconstructed TCGI but to 100% to evaluate the contribution of each terms at the TCGI change.

**Figure 5:**
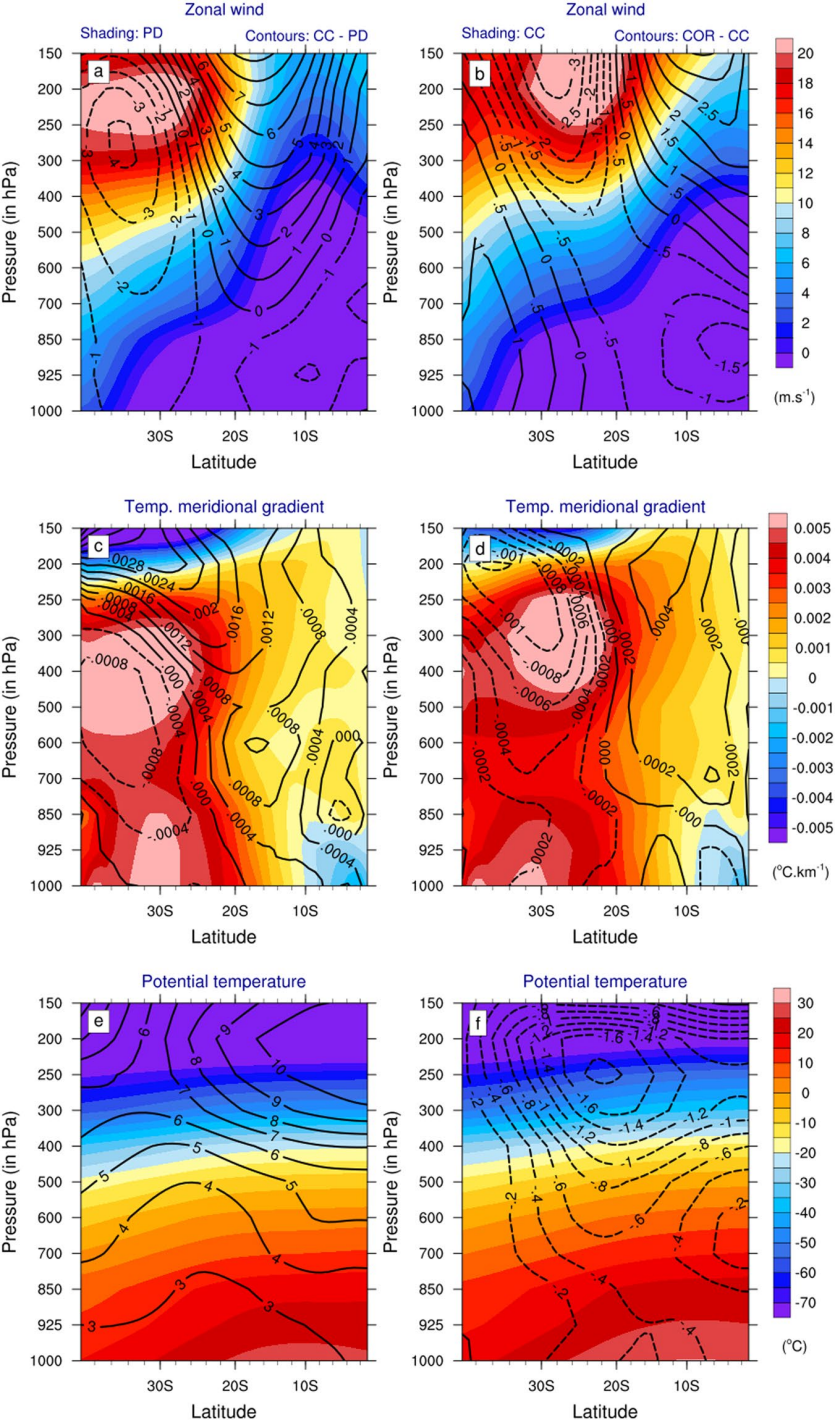
Colors show NDJFMA climatological vertical cross-sections zonally averaged over [145°E–230°E] of (top) zonal wind (in m.s^−1^), (middle) meridional temperature gradient (in °C.km^−1^) and (bottom) potential temperature (in °C) from (left) PD and (right) CC simulations. Contours show NDJFMA climatological vertical cross-sections zonally averaged over [145°E–230°E] of (top) zonal wind differences (in m.s^−1^), (middle) temperature meridional gradient differences (in °C.km^−1^) and (bottom) temperature differences (in °C) between (left) CC and PD and (right) COR and CC simulations. The dashed lines indicate negative values, while the solid lines indicate positive values.

Cyclogenesis indices allow to empirically assess the TC control by the large scale environment. Here we use the Tropical Cyclone Genesis Index (TCGI)^46^ to diagnose mechanisms responsible for the projected changes in TC activity through a Taylor’s expansion of the TCGI terms (see Methods section for a detailed description). This index reproduces the TC genesis decrease simulated by CC compared to PD, although it overestimates its amplitude (Fig. 4a). The Taylor’s expansion of TCGI change accurately reproduces the TCGI change. Figure 4a also indicates that the strengthening of vertical wind shear largely controls the weakening of TC genesis in CC compared to PD, this parameter accounting for ~85% of TC reduction. The zonal wind speed intensification at 200hPa is the dominant driver of the vertical wind shear strengthening in future conditions (not shown). As indicated by the contours in Fig. 5a, the tropospheric zonal wind increase from PD to CC is related to an intensification of the tropospheric westerlies on the equatorward side of the mid-latitude jet (north of 20°S) in the core of the SP cyclogenesis region. Most CMIP models also point towards such jet intensifications in the future^47^, which has been related to an increase in the upper tropospheric meridional temperature gradients^47^. In fact, because the atmospheric column warms more in the tropics than in the extra-tropics (contours in Fig. 5e), the upper tropospheric meridional temperature gradient from PD to CC increases equatorward of 20°S (contours in Fig. 5c) matching well with the zonal wind intensification in the upper troposphere (Fig. 5a). Our results hence indicate that such a mechanism also seems to operate in our simulations.

The larger TC number decrease in COR compared to CC (Fig. 3a vs 3b) can also be explained by the TCGI terms (Fig. 4b). This decrease is also largely driven by a strengthened vertical wind shear, accounting for ~70% of the TC number reduction (Fig. 4b). Indeed, correcting the projected CMIP SST warming pattern (COR vs CC experiments) leads to additional intensification the upper tropospheric westerlies and hence the vertical wind shear in the TC-prone region and weakens the jet south of 20°S (see contours in Fig. 5b). As between PD and CC, that change in zonal wind structure between CC and COR is consistent with changes in the meridional temperature gradient with an increased gradient north of 20°S and a decreased gradient to its south (contours in Fig. 5d). These temperature gradient changes responsible for upper tropospheric jets changes may be explained as follows: correcting the projected SST yields a larger drying of the SPCZ western portion as a consequence of near-surface south-easterlies penetrating further west and north in response to SST gradient changes, as already discussed in Dutheil *et al*.^26^ and Fig. 1b). This enhanced SPCZ drying results in an enhanced stratification of the temperature vertical profile south of 20°S (contours in Fig. 5f), which increases the meridional temperature gradient between the equator and 20°S in turn resulting in the upper level westerly anomalies (Fig. 5d).

## Discussion

In this study, we have evaluated the impact of climate change on TC activity using regional atmospheric simulations. Most existing studies use the PGW method to alleviate the current-day SST biases in CMIP models by adding the anomalous CMIP projected SST warming pattern to the observed present-day SST. Such method however does not eliminate in the anomalous warming pattern projected by these CMIP models such as those pointed out in Li *et al*.^8,25^. In the present study, we have tested the impact of correcting this warming pattern on the TC projections in the South Pacific, based on the statistical relation between CMIP present-day biases and projected SST changes as in Li *et al*.^25^.

Our results indicate that correcting the projected SST warming pattern considerably influences the projected changes in TC activity. While simulations forced with uncorrected CMIP5 SST warming pattern point towards a modest and insignificant decrease of TC number (−11%), simulations forced by the statistically-corrected SST warming pattern yield a far stronger and statistically significant decrease of the number of TCs (−55%). While our results are qualitatively consistent with most past studies, which also simulated a TC frequency reduction over the SP, the additional correction applied in our study has an impact which is as large as the range of TC reduction found in the existing literature, i.e. between 10%^17^ and 60%^43^. In other words, projected SST warming biases induce errors in TC changes in the SP as large as the combined uncertainties arising from the different model resolutions, convection schemes, scenarii and SST forcing patterns used in the previous studies addressing this topic. Therefore, it is crucial to correct the projected SST warming biases to improve the reliability of the SP TC projections.

Our results are consistent with past literature pointing out the strong sensitivity of regional TC projections to the projected SST warming patterns of different models for a given scenario^17–20^ even though this sensitivity has never been tested in an emergent constraint correction framework. For instance, Murakami *et al*.^18^ assessed the impact of the warming pattern on changes in TC activity by forcing their atmospheric model with three different classes of warming pattern projected by CMIP3 models. While their third cluster looks alike the ΔSST_CMIP_ pattern used in the present study, their second cluster (their Fig. 2c) is somewhat similar to our ΔSST_COR_ pattern applied (Fig. 1f), with a weaker western Pacific warming compared to the CMIP3 multi-model ensemble mean. Our results (i.e. stronger decrease of TC genesis when ΔSST_COR_ is applied compared to ΔSST_CMIP_) agree qualitatively with their analysis. Our results are also consistent with those of Knutson *et al*.^38^, which reveal a linear relationship between the regional SST change (relative to the tropical mean) and regional TC occurrence change, with the SP experiencing the strongest TC number decrease because it experiences the largest relative cooling.

Our results suggest that the changes in vertical wind shear is the main driver of the decrease in TC activity in our experiments irrespective of the projected SST correction strategy. This wind shear change can further be tracked back to a strengthening of the upper-tropospheric temperature meridional gradient in the cyclogenesis region. These results were obtained using the TCGI index. To assess their robustness, we compared them to those obtained with two other widely-used indices, namely the Genesis Potential Index (GPI)^48^ and its modified version (GPI*)^49^. This analysis confirms the dominant role of vertical wind shear, while the other terms respective contributions vary from one index to another (Fig. S2). The predominant role of changing vertical wind shear was also reported by Zhang and Wang^43^ using the GPI* index.

While our results indicate that correcting the projected SST biases strongly impacts the projected TC occurrence (and cyclogenesis), the impact is insignificant for other TC characteristics, such as the frequency of intense TCs or TC-related rainfall amount. Consistently with past literature^4,17,18,38,42,44,50^, our climate change simulations point toward a TC rainfall intensification (Fig. S3). Differences in the TC-related rainfall between the corrected and uncorrected experiments are however insignificant. The TC intensity distribution changes are also not substantially affected, with minor differences between the two future simulations (Fig. 3c).

The sensitivity of the future TC occurence reduction to the warming pattern may depend on the atmospheric model physical parameterizations and it may well be that the configuration discussed in this paper is particularly sensitive to SST gradients. To test this, we performed a similar set of simulations but with a completely different choice of physical parameterizations, including a different parameterization of subgrid-scale convection (details about this alternate configuration can be found in Table 1, see also^26^). While that other configuration is less realistic in simulating TC activity over the SP (Fig. 2 vs Fig. S4), changes in TC activity between the different future experiments are qualitatively similar in terms of TC genesis frequency changes (Fig. S5): the experiment forced by the corrected SST pattern indeed shows a larger decrease in TC count (−66%) than the uncorrected one (−39%). Changes in frequency of intense TCs in this set of experiments can however not be assessed because the alternative set of physical parameterisations does not allow to simulate the most intense TCs. That other set of simulations nonetheless highlights the robustness of our main result *i.e*. that future TC genesis projections in the SP are sensitive to the details of the projected SST pattern.

Aside from the model physics, our results may also be sensitive to the statistical method employed to correct the projected SST pattern. The underlying assumption with the PGW approach is the stationarity of the biases between the present-day and the future conditions. This hypothesis is strongly challenged by recent studies^24,25^ showing a modification of the biases. Here, we use the same method as in Li *et al*.^25^ where the SST projection is corrected based on the western equatorial Pacific present-day rainfall bias. This method uses a simple linear relationship that emerges from the CMIP5 models spread. The linear relationship is probably the strongest assumption of that method. Indeed, the relationship is not perfectly linear for all locations (r = −0.57). Nevertheless, using a different observational constraint method, Huang and Ying^24^ also show a very similar corrected SST pattern (their Figure 11c) to the one inferred by Li *et al*.^25^ (their Fig. 12c), and used in the present study (Fig. S10c). Despite such encouraging robustness these physically-grounded methods aiming at providing a more realistic regional projections are still in their infancy and probably deserve more research. For instance, the TC projections in our bias-corrected simulation may be sensitive to the changes applied to the lateral boundary conditions. We did not apply the same type of correction to the atmospheric lateral boundaries because contrary to the projected SST change, which displays a clear statistical relation with the dry equatorial bias, we did not find robust statistical relations between the lateral boundary conditions projected changes and the dry equatorial bias. We hence could not correct the projected CMIP multi-model mean lateral boundary changes using the method of Li *et al*.^25^. We did however test the sensitivity of our results to changes in the lateral boundary conditions by applying those of the ACCESS1-0 model^51^ instead of the CMIP5 multi-model mean. We chose this particular model because its projected SST change was very similar to that applied in the COR experiment (Supplementary Information Figs. S6–7), ensuring a reasonable physical consistency between the applied SST and lateral boundary condition changes. The projected Southwestern Pacific TC changes were shown to be unsensitive to changes in lateral boundary conditions between that experiment and COR (1.8 TCs.year^−1^ in COR against 1.7 TCs.year^−1^ in ACCESS1-0, Supplementary Information Fig. S9). This unsensitivity to lateral boundary conditions may be related to the fact that the lateral boundary conditions (at 42°S, 26°N, 101°E and 59°W) in our experimental setup are quite far from the Southwest Pacific nested domain over which we examine the TC projections (32°S to 2°S, 145°E to 130°W).

Our study indicates that the projected SST change is a primary source of uncertainty for TC activity changes projections under climate change in the SP. Due to computational constraints, we however did not test if our correction strategy resulted in a reduction of the uncertainties in TC projections. Additional experiments forced by uncorrected and corrected SST changes from several individual CMIP5 models should be carried out to ascertain if this strategy leads to a reduction of the TC projection uncertainties and hence to an improved reliability of TC projections in this region. In any case, our results suggest that atmospheric simulations using a PGW downscaling approach are very sensitive to this SST pattern correction. Future studies should apply similar strategies using different models, alternative observational constraints, and in other TC-prone regions in order to further test if the sensitivity of projected TC activity changes to details of the projected SST changes is ubiquitous.

## Methods

### The emergent constraint approach to correct the projected SST field

In this study, the SST response projected by CMIP5 models is corrected based on the present-day rainfall bias in the western equatorial Pacific using an emergent constraint method. A regional climate model is then used to test the sensitivity of projected TC activity to this corrected SST change. The correction method is basically the same as the one proposed by Li *et al*.^25^. The annual SST climatology for present-day (last 20 years, *i.e*., 1989–2009) and the future (last 20 years; 2080–2099) scenario are respectively denoted by *SST*^*PD*^*(s,m)* and *SST*^*CC*^*(s,m)*, where ‘*s*’ represents space (longitude-latitude) coordinates and ‘*m*’ model IDs. The SST response to climate change for each model can then be calculated as the difference between the future and historical SSTs as:

1 $$\varDelta SST(s,m)=SS{T}^{CC}(s,m)-SS{T}^{PD}(s,m)$$

The present-day annual average rainfall bias in the western equatorial Pacific (WEP; 140°E–170°W; 2°S–2°N) is computed as the difference between the 1989–2009 average rainfall in present-day CMIP simulations and observations (CMAP) as follows:

2 $$P{r{\prime} }_{WEP}(m)=P{r}_{WEP}^{PD}(m)-P{r}_{WEP}^{OBS}$$

where $P{r{\prime} }_{WEP}\,(m)$ is the annual rainfall bias averaged over the WEP for the model *m*, $P{r}_{WEP}^{PD}(m)$ is the annual rainfall averaged over the WEP for the model *m* in CMIP present-day simulations, and $P{r}_{WEP}^{OBS}\,$is the annual rainfall averaged over the WEP for observations, all obtained for the 1989–2009 period.

The relationship between the projected SST changes and the WEP present-day rainfall bias can be obtained through a linear regression analysis as,

3 $$\varDelta SST(s,m)=R(s)\ast P{r{\prime} }_{WEP}(m)+res(s,m)$$

where *R(s*) is the spatially-dependent regression coefficient and *res*(*s, m*) is the residual, which represents the projected response of climate change at zero bias, *i.e*. as the corrected response (*ΔSST*_*COR*_) to climate change for each model:

4 $$\varDelta SS{T}_{COR}(s,m)=res(s,m)$$

The multi-model mean of the corrected response to climate change is calculated as:

5 $$\varDelta SS{T}_{COR}^{MMM}(s)=\frac{1}{N}\mathop{\sum }\limits_{m=1}^{n}\varDelta SS{T}_{COR}(s,m)$$

where *n* is the total number of models. Figure S10 displays the MMM of all terms of the Eq. 3.

### Regional model configuration and experimental design

As in Dutheil *et al*.^26^, we use the Weather Research and Forecasting Model version 3.6.1^52^ with a parent domain at 105 km resolution that encompasses the tropical Pacific region [101°E–59°W; 26°N–42°S]. To simulate the South-West (SW) Pacific cyclogenesis, we add a two-way nested domain located over the SW Pacific [145°E–130°W; 32°S–2°S] with an increased resolution of 21 km. Both domains share the same 32 vertical levels in terrain-following coordinates. The main configuration discussed here includes Lin *et al*.^53^ microphysics scheme, the Community Atmosphere Model^54^ parameterizations for shortwave and longwave radiation, the University of Washington planetary boundary layer scheme^55^ with the Monin–Obukhov surface layer parameterization, the Noah land surface model^56^, and the Zhang and McFarlane^57^ parameterization for subgrid-scale convection. This model reproduces observed TC characteristics reasonably well in this region^43,58–60^. To evaluate the robustness of the results obtained from this configuration, we compare them to those obtained from another configuration using a completely different model physics in the last section. The two physical configurations are the same as those used in Dutheil *et al*.^26^, and are further detailed in Table 1.

**Table 1:** Physics parametrization selected in the ZM and BMJ model configurations.

Configuration	ZM	BMJ
Deep convection	Zhang & McFarlane^57^	Janjic^63^
Microphysic	Lin *et al*.^53^	Hong *et al*.^64^
Longwave and shortwave radiation	Collins *et al*.^54^	Iacono *et al*.^65^
Planetary boundary layer	Bretherton & Park^55^	Noh *et al*.^66^
Land-surface model	Chen & Dudhia^56^	Chen & Dudhia^56^
Oceanic surface layer	Jiménez *et al*.^67^	Jiménez *et al*.^67^

A present-day simulation (labelled PD) is first performed over the 1980–2016 period (37 years). Surface and lateral boundary conditions for the parent domain are taken from 6-hourly outputs of the NCEP2 reanalysis^61^. Two future simulations are conducted by prescribing anomalies for surface (SST) and lateral (wind, temperature, specific humidity, and geopotential height) boundaries from the multi-model mean CMIP5 projections under the RCP8.5 scenario in the late twenty-first century. This strategy ensures that the specified change in boundary conditions obeys the same linear balances (*e.g*. geostrophy, thermal wind…) than the individual general circulation models from CMIP5, and that the SST and lateral boundary precipitation changes are thus physically consistent. For those, initial, surface, and lateral boundary conditions are taken as the sum of NCEP2 reanalysis and the mean seasonal cycle of future projected changes computed as the ensemble mean of monthly climatology difference of late twenty-first-century (2080–2099) and historical simulations (1989–2009) of 31 CMIP5 models. Hence, the synoptic and interannual variability at the boundaries keeps the same phase and amplitude as the PD simulation. The two future simulations only differ through the applied pattern of projected sea surface warming. In the first one (labelled CC), the uncorrected CMIP5 ensemble-mean SST warming pattern (ΔSST_CMIP_) is used. In the second one (labelled COR), the CMIP5 ensemble-mean SST pattern is statistically corrected (ΔSST_COR_) as described in the emergent constraint approach.

### TC tracking method

We use a tracking method to detect projected change in TCs, by comparing cyclones in various present-day and future simulations. The tracking method used here was developed by Chauvin *et al*.^62^, and previously used in similar WRF configurations to that in the present study^58,60^. The following criteria are used to distinguish tropical cyclones from intense mid-latitude systems at each output time step (6 h):

- local minimum in sea level pressure;
- 850 hPa vorticity > VOR;
- maximum 850 hPa wind speed > WT;
- Mean 700-300 hPa temperature anomaly > TT;
- 300 hPa temperature anomaly > 850 hPa temperature anomaly;
- 850 hPa tangential wind > 300 hPa tangential wind.

VOR, WT and TT are threshold parameters for vorticity, wind speed and temperature anomaly respectively. The thresholds retained for this study are: VOR = 20.10^–5^s^−1^; WT = 17 m.s^−1^ and TT = 1 K. The VOR and TT thresholds are empirical and configuration-dependent, and were chosen using a similar approach as in Jourdain *et al*.^58^. A positive TT criterion is important to detect warm core vortices, whereas excessive values fail to detect all tropical cyclones. The vorticity threshold has a significant role in filtering weaker mesoscale vortices. Moreover, we have removed TCs with a shorter lifetime than 2 days. Importantly for this study, the tracking method does not depend on SST or absolute air temperature, but rather on temperature anomalies, so that the number of detected TCs is not affected by a uniform tropospheric warming.

The modelled TC activity in the PD simulation is compared to the TC activity inferred from the latest version (v03r09) of the International Best Track Archive for Climate Stewardship (IBTrACS) database^57^ over 1980–2016.

### TC diagnostics

TGCI is a cyclogenesis index built from 4 environmental parameters, which can be written as follows:

6 $$TGCI=exp({b}_{\eta }\eta )\ast exp({b}_{Vs}.Vs)\ast exp({b}_{HR}.HR)\ast exp({b}_{T}.T)\ast exp(b+log(cos\,\Phi ))$$

where η is the absolute vorticity at 850hPa (s^−1^), HR the relative humidity at 600 hPa (%), T the relative SST (in °C) and Vs the magnitude of the vertical wind shear between 850 and 200 hPa (m.s^−1^). Ф represents the latitude. As in Camargo *et al*.^62^, The coefficients (${b}_{\eta },{b}_{Vs},{b}_{HR},{b}_{T},b$) are obtained as a result of the best fit of the multi-linear regression with the simulated climatological TC genesis in PD using the environmental parameters derived from the PD simulation. In a similar way as in Camargo *et al*.^62^, this ensures that the TGCI is fitted to capture the cyclogenesis climatology simulated in the PD simulation.

Following Zhang and Wang^42^, the contribution of each factor to TC genesis changes are derived from a Taylor’s series expansion of the TCGI terms. There are four terms including an environmental parameter in the TGCI formula. The contribution of each of these four terms to the future TGCI change is estimated by individually varying each term as:

7 $$\varDelta TGC{I}_{i}=\varDelta {T}_{i}\ast ({T}_{j1}\ast {T}_{j2}\ast {T}_{j3}\ast exp(b+log(cos\,\Phi )))$$

8 $${\rm{with}}\,\varDelta TGCI\approx \mathop{\sum }\limits_{i=1}^{4}\varDelta TGC{I}_{i}$$

where Δ represents the future change (*i.e*. CC or COR minus PD), the subscript *i* being the contributing term and the subscript *j*1 to *j*3 being the remaining terms in PD. As shown in Fig. 4, the reconstructed ΔTGCI is relatively close to the actual ΔTGCI, demonstrating the validity of the reconstruction. To assess the sensitivity of our results to index selection, we compared the TCGI with two others cyclogenesis index (Fig. S1), the GPI and GPI* defined in supplementary document.

### Statistical testing

We used a Mann–Whitney–Wilcoxon test to check if the TC activity (genesis, occurrence, and intensity) differences between the PD and future simulations are statistically significant. This test is the non-parametric equivalent to the independent *t*-test. It is used to test whether two samples are likely to derive from the same population, without making any assumption about the distribution of data contrary to a *t*-test. Our null hypothesis is that PD and the future simulations have the same climatological mean value. We applied the test to the time series of the TC genesis and occurrence annual means.

## Supplementary information

Supplementary information.
