# Supplementary material for: Impact of projected sea surface temperature biases on tropical cyclones projections in the South Pacific
Source: Sci Rep. 2020 Mar 16;10:4838. doi: 10.1038/s41598-020-61570-6 (PMC7075914; doi:10.1038/s41598-020-61570-6)
Supplement: Supplementary file 1 — Supplementary information. [file 41598_2020_61570_MOESM1_ESM.docx]

Supporting Information for

**Impact of projected sea surface temperature biases on tropical cyclones projections in the South Pacific**

Cyril Dutheil^1^, Lengaigne M.^2^, Bador M.^3^, Vialard J.^2^, Lefevre J.^1^, Jourdain N.^4^, Jullien S.^5^, Peltier A.^6^, Sultan. B^7^ and Menkes C^8^

^1^ IRD (Institut de Recherche pour le Développement)-Sorbonne Universités (UPMC, Université Paris 06)-CNRS-MNHN-IPSL,  LOCEAN Laboratory, IRD Nouméa BP A5, 98848 Nouméa cedex, New Caledonia.

^2^ LOCEAN-IPSL, Sorbonne Universités, UPMC, Université Paris 06, CNRS-IRD-MNHN, Paris, France.

^3^ Climate Change Research Centre and ARC Centre of Excellence for Climate Extremes, School of BEES, University of New South Wales, Sydney, New South Wales, Australia.

^4^ Univ. Grenoble Alpes, CNRS, IRD, G-INP, IGE, Grenoble, France

^5^ Ifremer, Univ. Brest, CNRS, IRD, Laboratoire d’Océanographie Physique et Spatiale (LOPS), IUEM, Plouzané, France

^6^ Météo France, Nouvelle Calédonie

^7^ ESPACE-DEV, Univ Montpellier, IRD, Univ Guyane, Univ Reunion, Univ Antilles, Univ Avignon, France

^8^ IRD, ENTROPIE (UMR 9220), BP A5, 98848 Nouméa cedex, New-Caledonia

**Contents of this file**

Figures S1 to S10

**Figure S1.** Composite of the probability distribution functions (PDFs) of TC genesis anomalies in the observations (left column; IBTrACS) and in PD simulation (right column) for El Nino (first row) and La Nina (2^nd^
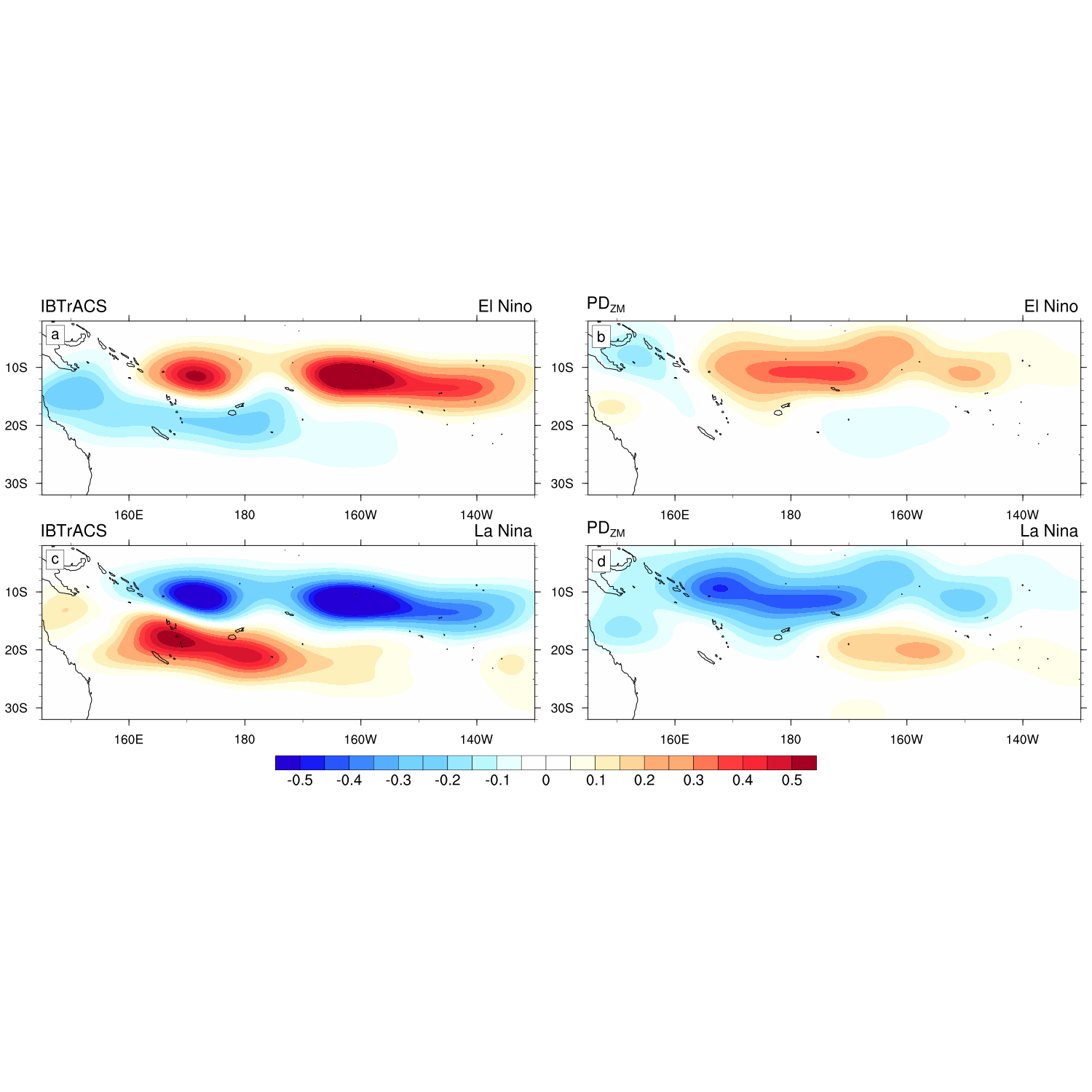
 row) phases. To generate PDFs, we compute anisotropic Gaussian functions, with an associated standard deviation in meridional and zonal directions respectively of 2.5° and 5°.

To assess the sensitivity of our results to index selection, we compared the TCGI with two others cyclogenesis index (Fig. S2), the GPI and GPI* defined as follows:

$GPI=\left| {10}^{5}\eta\right|^{3/2}\left( \frac{RH}{50} \right)^{3}\left( \frac{V_{pot}}{70} \right)^{3}\left( 1+0.1V_{s} \right)^{-2}$

and,

${GPI}^{☼}=\left| {10}^{5}\eta\right|^{3/2}\left( \frac{RH}{50} \right)^{3}\left( \frac{V_{pot}}{70} \right)^{3}\left( 1+0.1V_{s} \right)^{-2}\left( \frac{-\omega+0.1}{0.1} \right)$,

where η is the absolute vorticity at 850hPa (s^-1^), RH is the relative humidity at 700hPa (%), V_pot_ is the maximum potential intensity (m.s^-1^), V_s_ is the magnitude of the vertical wind shear between 850 and 200 hPa (m.s^-1^), and ω is the vertical wind velocity (Pa.s^-1^).

*
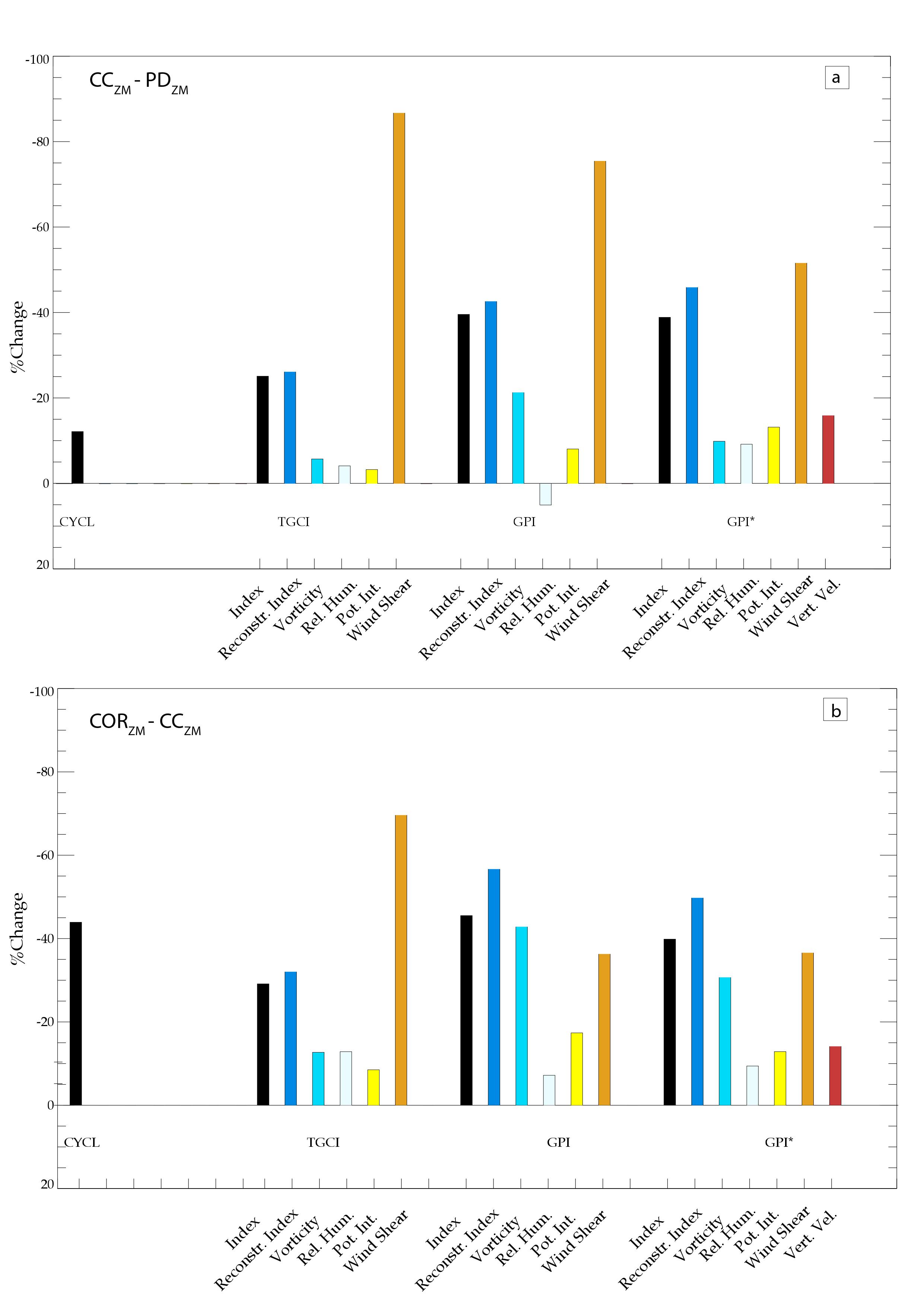
*

**Figure S2:** Same as figure 3 but for the 3 TC genesis indices: TCGI, GPI and GPI*. In GPI* the contribution of vertical wind velocity at 500hPa is also evaluated (red bar).


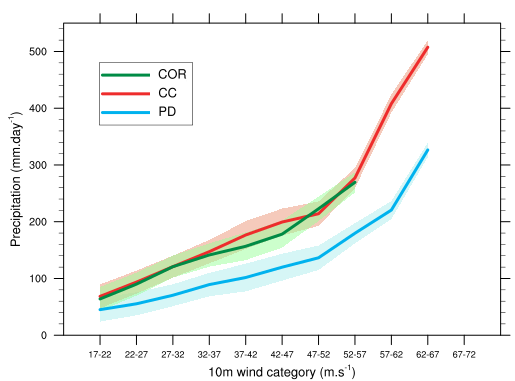


**Figure S3.** Precipitation under TCs (in mm.day^-1^) as a function of the 10m wind speed category (in m.s^-1^) for PD (blue), CC (red) and COR (green) simulations.


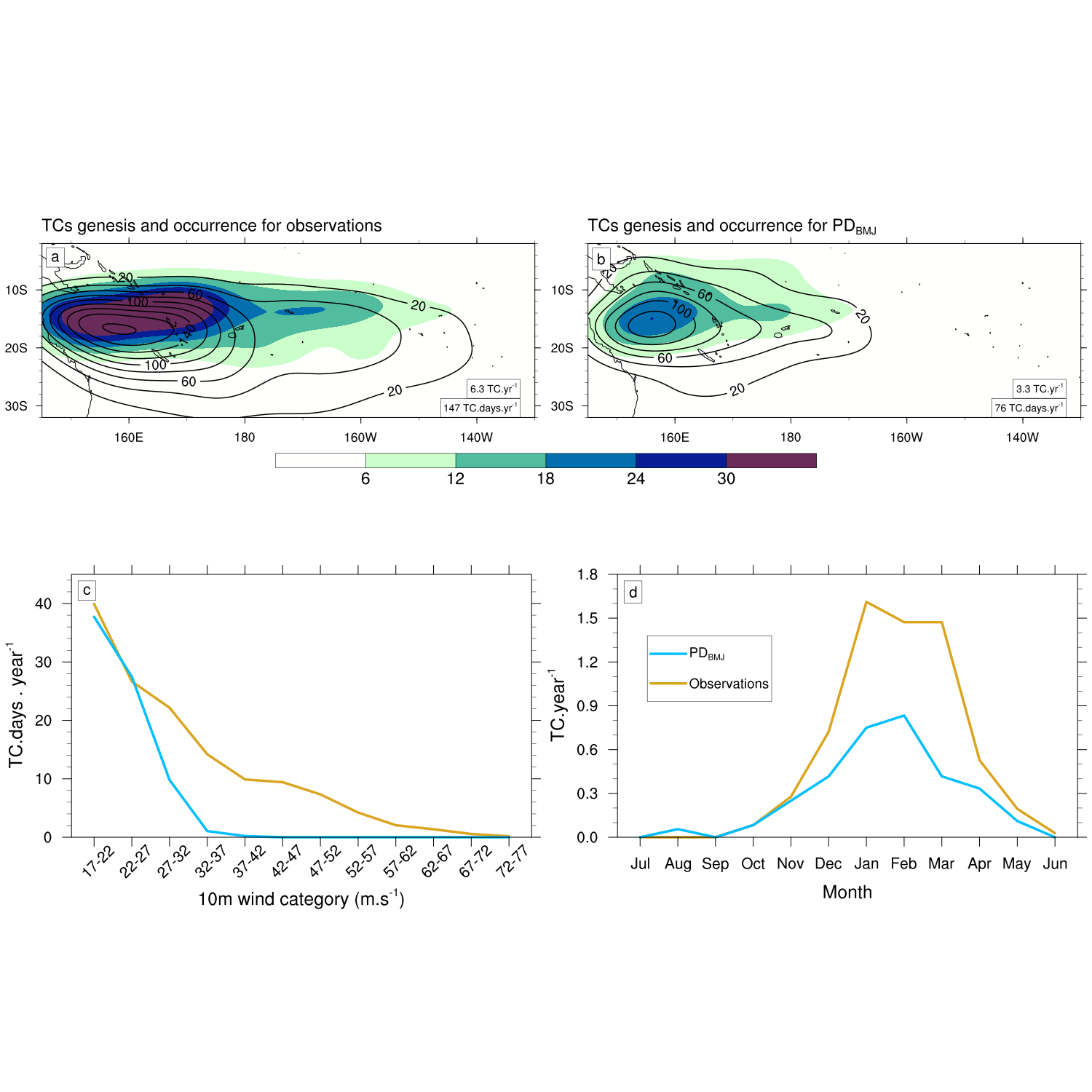


**Figure S4.** Top: Probability Density Function of TC genesis (shading) and occurrence (contour lines) for a) observations (IBTrACS), and (b) PD_BMJ_ simulation. The annual mean TC genesis and occurrence are annotated in the corresponding panel. Bottom: (c) Annual mean frequency of TC occurrence (in TC.days .year^-1^) as a function of the maximum 10-m wind speed (in m.s^-1^) and (d) the seasonal cycle of monthly TC genesis number (in TC.year^-1^) for observations (gold) and PD_BMJ_ (blue) simulation.


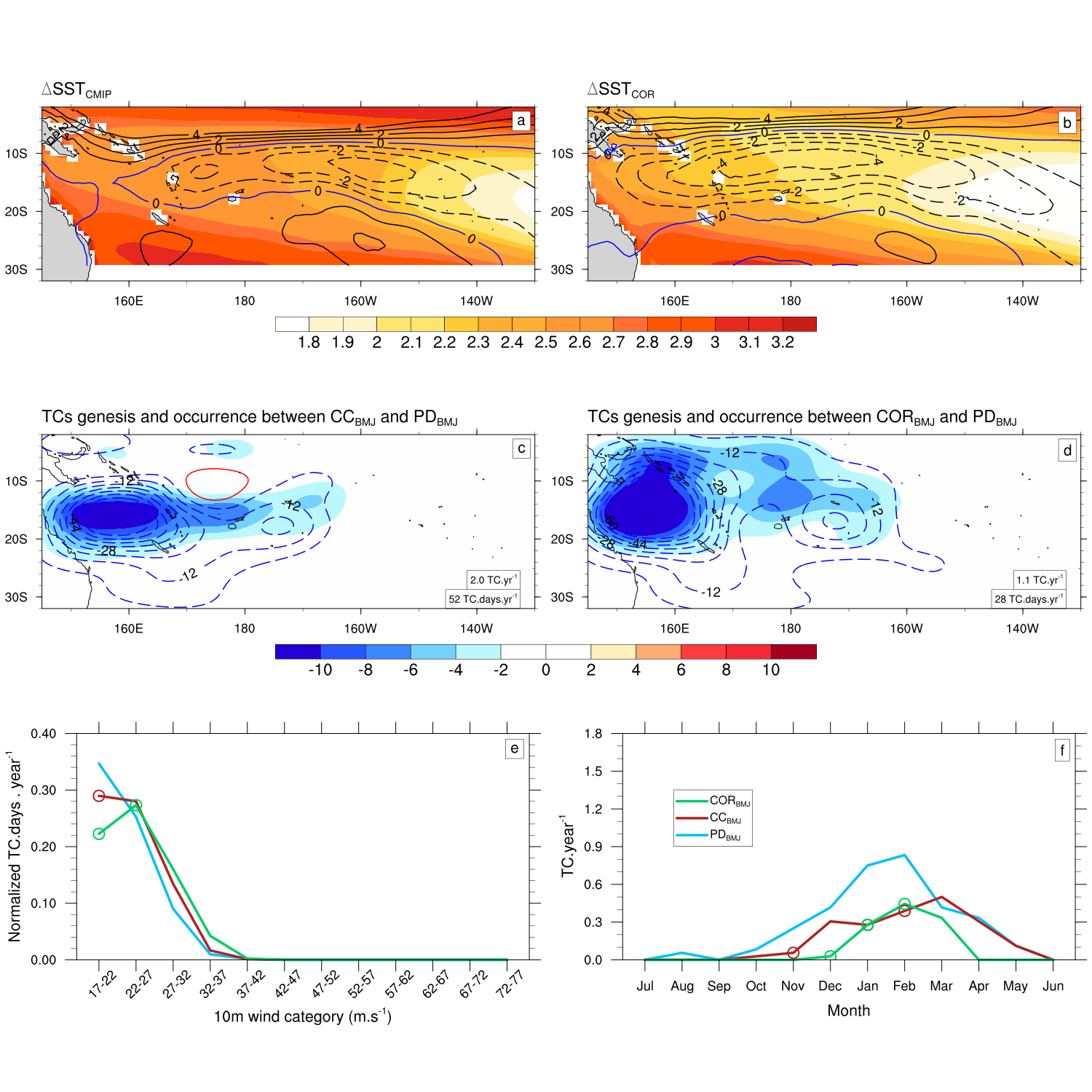


**Figure S5.** Top: DJF climatology (shading, in °C) of (a) ΔSST_CMIP_ and (b) ΔSST_COR_. The contours represent the precipitation changes (in mm.d^-1^) between (a) CC_BMJ_ and PD_BMJ_, (b) COR_BMJ_ and PD_BMJ_ simulations. The dashed lines indicate negative values, and the thick lines indicate positive values. Middle: Probability density functions of TC genesis (shading) and occurrence (contour lines) between (c) CC_BMJ_ and PD_BMJ_ and (d) COR_BMJ_ and PD_BMJ_ simulations. The values of annual mean TC genesis and occurrence are annotated in the corresponding panel. Bottom: (e) Annual mean frequency of TC occurrence (in TC.days .year^-1^) as a function of the maximum 10-m wind speed (in m.s^-1^) and (f) the seasonal cycle of monthly TC genesis number (in number of TC.year^-1^) for PD_BMJ_ (blue), CC_BMJ_ (red) and COR_BMJ_ simulations.


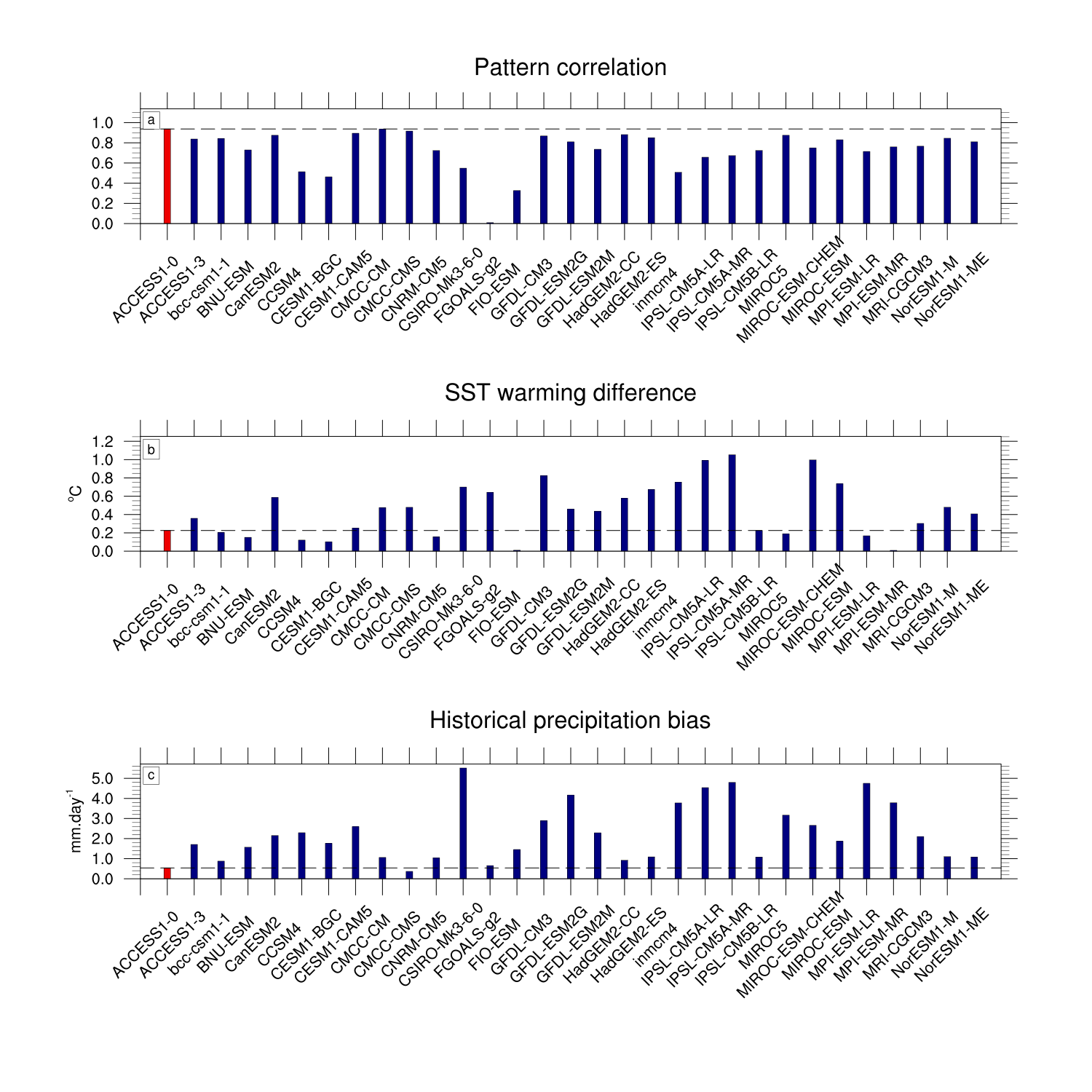


**Figure S6.** Barplots of three metrics used to select the model for the lateral boundary condition sensitivity experiment: (a) pattern correlation between ΔSST_COR_ and ΔSST of each CMIP5 model over the entire domain; (b) difference between the area-average of ΔSST_COR_ and ΔSST of each CMIP5 model (in °C); (c) precipitation difference (in mm.day^-1^) between CMAP observations and each CMIP5 model in the Western equatorial Pacific [160°E-170°W;2°S-2°N]. For the SST warming difference and the historical precipitation bias (panels b and c), absolute values are displayed to facilitate comparison. On each panel, the red bar and dashed line shows the value of ACCESS1-0 model, which has been selected for our sensitivity test.


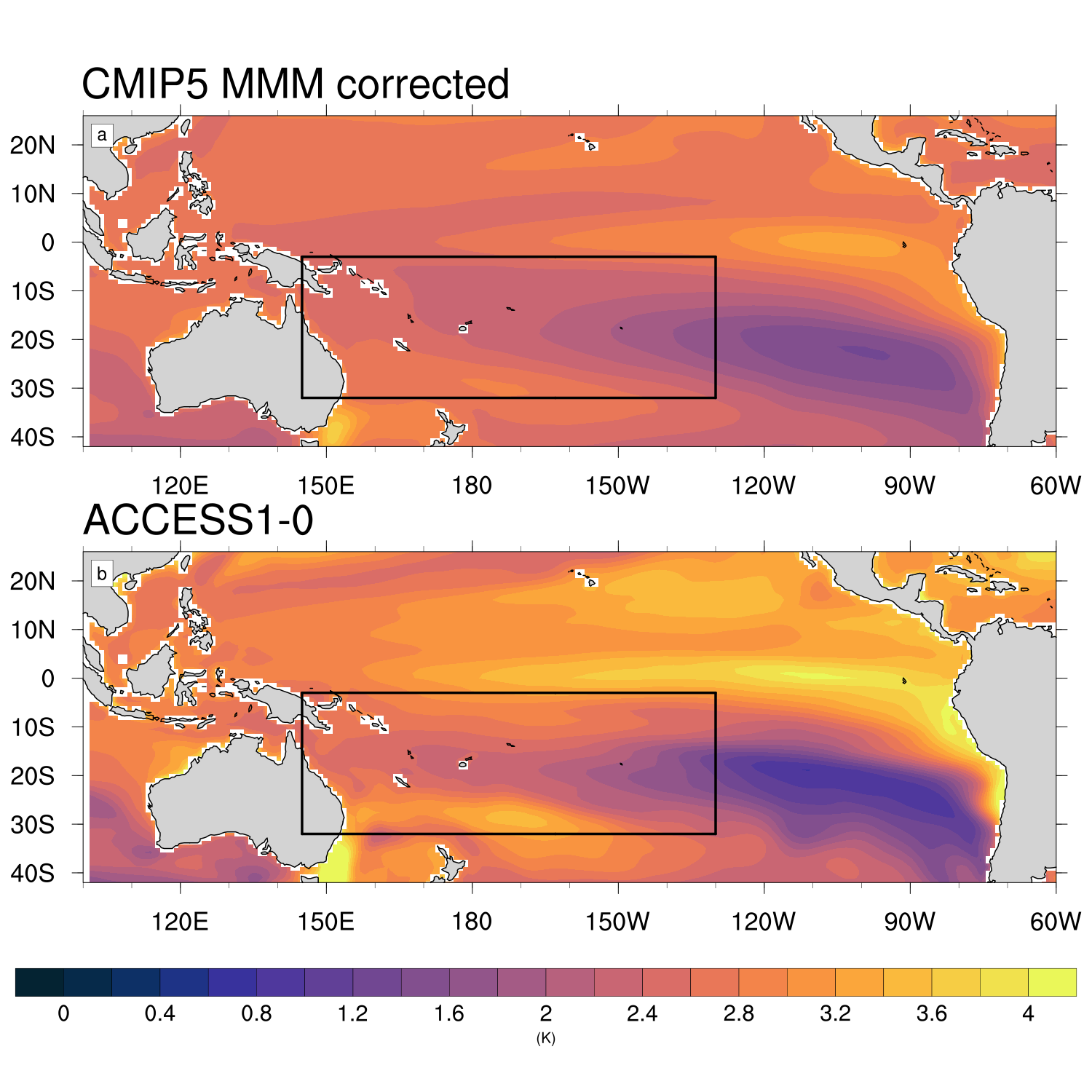


**Figure S7.** Annual climatology of SST warming pattern (in °C) for (a) ΔSST_COR_ and (b) ΔSST_ACCESS1-0_. The black box on each panel represents the nested domain [145°E-130°W;32°S-2°S] over which the TCs are simulated.

**Figure S8.** Annual climatology of the difference between ACCESS1-0 model and the CMIP5 MMM at each lateral boundary (west, east, south and north) for (left) air temperature (in °C) and (right) specific humidity (in kg_water_/kg_moist air_). Contours represent the projected changes for the CMIP5 MMM.
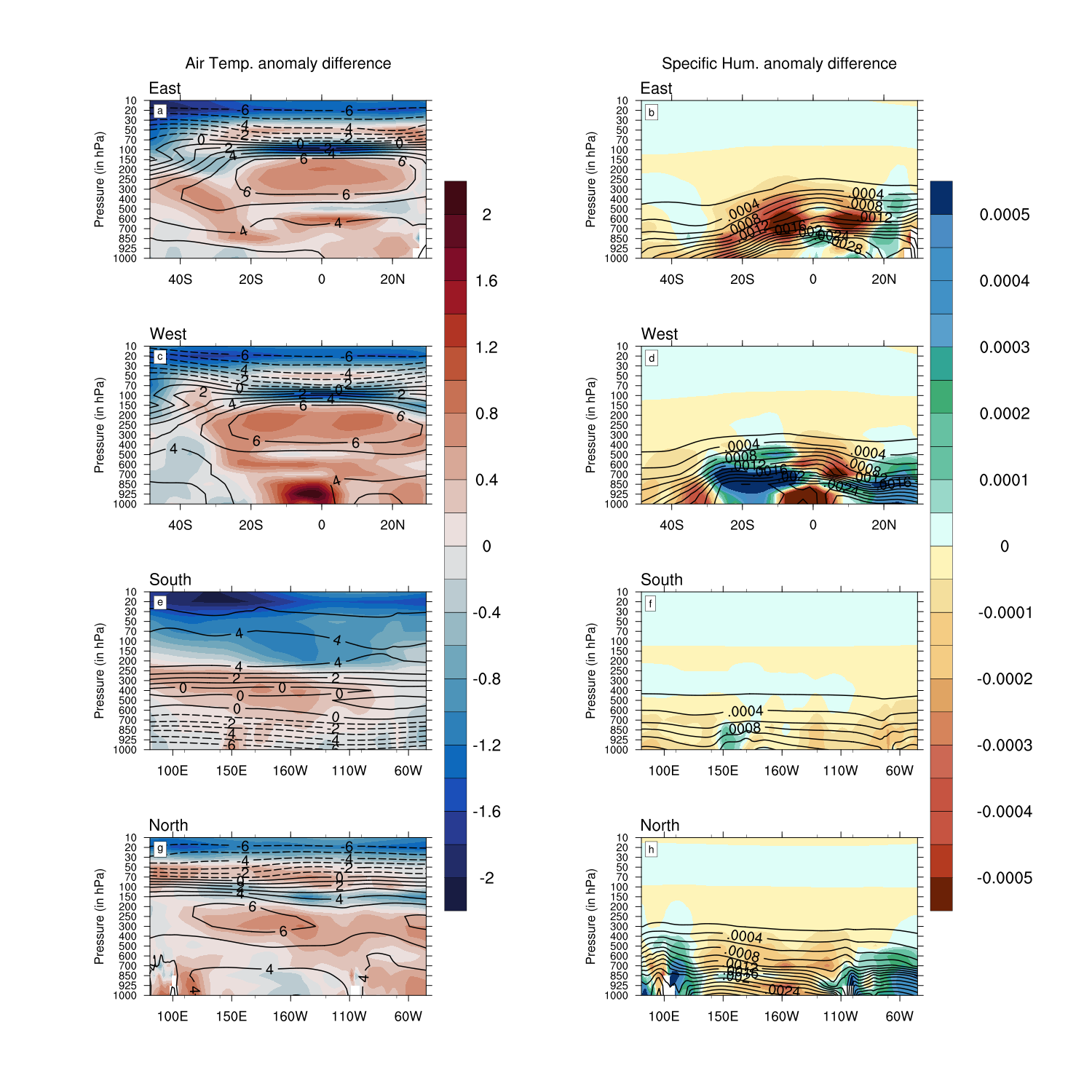


Our TC projections in our bias-corrected simulation may be sensitive to the changes applied to the lateral boundary conditions. While we applied a correction to the projected SST change based on the existing statistical relation with the dry equatorial bias, we indeed could not apply the same type of correction to the atmospheric lateral boundaries because there no robust statistical relationship between the lateral boundary conditions projected changes and the dry equatorial bias. To test the sensitivity of our results to the lateral boundary conditions applied, we did a sensitivity experiment where we applied the lateral boundary conditions from the ACESS1-0 model instead of the CMIP5 MMM in the COR experiment. We did select this specific CMIP5 model because it has a projected SST change that is closest to the bias-corrected MMM SST projection. To identify that model, we indeed calculated three indices evaluating how close the projected SST change is from the MMM corrected SST change (pattern correlation and domain-averaged difference, Figure S6a,b), and how small the present-day precipitation bias is (domain averaged precipitation bias, Figure S6c). ACCESS1-0 has one of the closest projected SST change to that of the “COR” experiment, with a SST pattern correlation of 0.94 (1^st^ rank) and SST difference of 0.23°C (10^th^ rank). It also has one of the smallest present-day precipitation biases (0.53mm.d^-1^, 2^nd^ rank). The fact that ACCESS1-0 displays a projected SST change (Figure S7b) that is close to the corrected MMM SST change (Figure S7a) ensures that its boundary conditions are more physically consistent with the corrected MMM SST change than those of the CMIP5 MMM. As shown on Figure S8, those lateral boundary conditions deviate from the CMIP5 MMM by up to +/- 1.5°C for temperature and 0.0005 kg_water_/kg_moist air_ for specific humidity (i.e. +/-30% relative changes for both variables).

We thus performed a 10-year climate-change simulation where we applied ΔSST_COR_ at the surface and projected lateral boundary changes from ACCESS-1-0 model instead of the CMIP5 MMM, to test the sensitivity to lateral boundary conditions. As illustrated on Figure S10, our results indicate that the projected change in the TCs number is insensitive to the change of lateral boundary conditions (1.8 vs 1.7 TC.year^-1^), the spatial pattern being also very similar between the two experiments. I.e. the projected change in southwest Pacific TCs number is much less sensitive to changes in lateral boundary conditions than to correcting SST using the method of Li et al. (2016). This weak sensitivity to lateral boundary conditions is likely related to the fact that the lateral boundary conditions (at 42°S, 26°N, 101°E and 59°W) in our experimental setup are quite far from the southwest Pacific nested domain over which we examine the TC projections (32°S to 2°S, 145°E to 130°W).


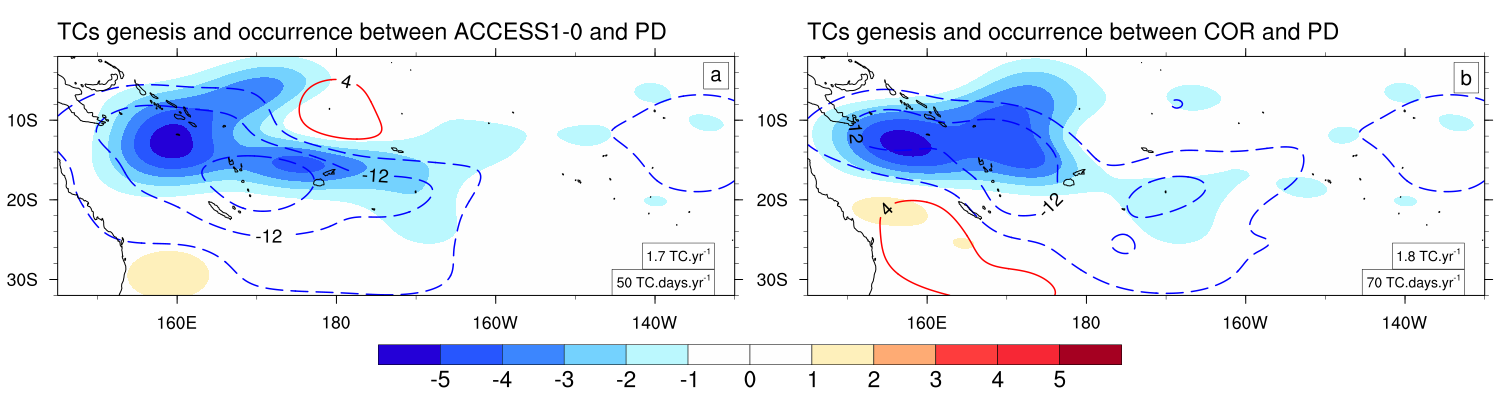


**Figure S9.** Probability Distribution Functions (PDFs) of TC genesis (shading) and occurrence (contour lines) computed over the 1980-1990 period between (a) PD and COR and (b) PD and ACCESS1-0 (which is an experiment similar to COR except that projected changes in the lateral boundary conditions are those from ACCESS1-0 model instead of CMIP5 MMM). The values of annual mean TC genesis and occurrence in COR and ACCESS1-0 are shown in the corresponding panels.


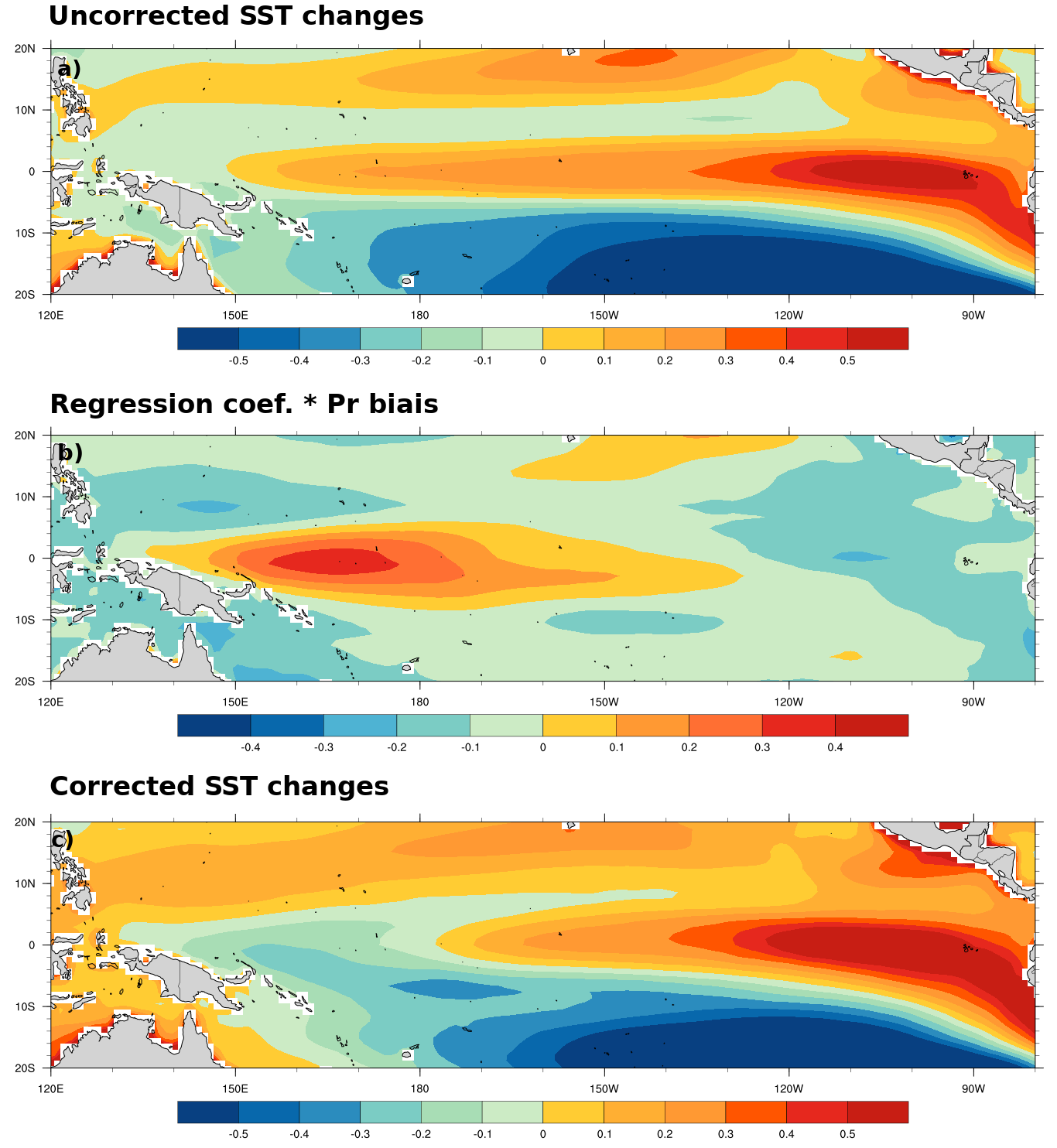
**Figure S10:** Multi model mean of all terms of the equation 3, (a) $\Delta SST\left( s \right)$, (b) $R\left( s \right)*Pr'_{WEP}$ and (c) res(s). To highlight the spatial pattern, the tropical Pacific mean warming of SST for each model is removed in a–c.
